# Investigating milk-derived extracellular vesicles as mediators of maternal stress and environmental intervention

**DOI:** 10.1038/s41380-025-03346-w

**Published:** 2025-11-17

**Authors:** Julia Martz, Baila Hammer, Tristen J. Langen, Benjamin N. Berkowitz, Benzion Berkowitz, Jasmyne A. Storm, Jueqin Lu, Deepali Lehri, Sanoji Wijenayake, Jordan Marrocco, Amanda C. Kentner

**Affiliations:** 1 School of Arts & Sciences, Health Psychology Program, Massachusetts College of Pharmacy and Health Sciences, 179 Longwood Avenue, Boston, 02115 Massachusetts USA; 2 Department of Biology, Touro University, 3 Times Sq, New York, 10036 NY USA; 3 Department of Biology, The University of Winnipeg, 515 Portage Ave, Winnipeg, Manitoba Canada; 4 Genomics Core Laboratory, New York Medical College, 15 Dana Road, Valhalla, 10595 NY USA; 5 Department of Physiology, New York Medical College, 15 Dana Road, Valhalla, 10595 NY USA; 6 Feil Family Brain and Mind Research Institute, Weill Cornell Medicine, 1300 York Ave, New York, 10065 NY USA

**Keywords:** Neuroscience, Molecular biology, Psychology, Cell biology, Biomarkers

## Abstract

Parental communication signals are transmitted through nursing and critically shape neurodevelopmental trajectories. Mirroring some well characterized effects of gestational challenges in rodents, maternal immune activation (MIA) during the lactational period disrupts maternal physiology, decreases lipid content in milk, and is associated with adverse neurobehavioral outcomes in offspring. This occurs without MIA significantly affecting maternal care. While gestational MIA models are responsive to environmental interventions, which beneficially alter maternal milk composition and associated offspring outcomes, the bioactive mediators in milk underlying resilience remain poorly understood. Milk-derived extracellular vesicles (MEVs) transport and deposit biologically active cargo, including microRNAs (miRNAs) that induce post-translational regulation of candidate mRNA, in the nursing offspring’s tissues and cells. Using a rat model, we show that lactational MIA alters MEV-miRNA cargo and the expression of miRNAs in offspring hippocampus. Several miRNAs in MEVs were also found in the hippocampus of matching offspring. Remarkably, the miRNA changes in MEVs and the neonatal hippocampus were rescued when dams were raised in an enriched environment, suggesting environmental enrichment protected from the effects of MIA. This was supported by the behavioral phenotype. RNA-seq of adult offspring hippocampus showed long-term transcriptional changes associated with the gene targets of early-life regulated miRNAs. Our results position MEV-miRNA as dynamic programming signals by which maternal experience is communicated to offspring, encoding both stress-induced and protective cues that influence development. This suggests that breastfeeding interventions can regulate the genetic cargo of the milk, programming the life of developing infants.

## Introduction

Intergenerational transmission of biological cues shape offspring neurodevelopment and long-term behavioral outcomes. Beyond direct caregiving and nutritional transfer through nursing, recent research has identified extracellular vesicles (EVs) as a mechanism through which parental signals may be transmitted to offspring [1–7]. EVs are membrane-bound nanovesicles containing microRNAs (miRNAs), messenger RNAs (mRNAs), proteins, peptides, and lipids, capable of influencing gene expression and cellular function in target tissues [8, 9]. While the mechanisms underlying parental contributions to offspring development via reproductive tract-derived EVs and sperm are well described [2, 3], maternal EV signaling during lactation remains less understood.

Breast milk is a biologically active medium; it supplies nutrition and other regulatory information to offspring [10]. Indeed, breastfeeding is associated with numerous benefits for infant development, including enhanced immune function, cognitive outcomes, and reduced risk for metabolic diseases [10–13]. Milk contains a substantial population of milk-derived extracellular vesicles (MEVs) that can survive the digestive tract, enter systemic circulation, and reportedly accumulate in peripheral tissues and the brain, including the hippocampus of nursing neonatal mice [5, 6, 14–19]. Complementary evidence from a blood brain barrier (BBB) cell-based model demonstrates that peripherally derived EVs transport their cargos across brain microvascular endothelial cells. This is most apparent under inflammatory conditions when barrier integrity is reduced [20]. Moreover, although the BBB is established in neonates, some brain associated barriers are more permeable to small molecules during this developmental stage [21–23], a time when the brain shows increased bioavailability to peripherally administered EVs compared to adults [24]. These findings highlight that early life contexts characterized by reduced BBB permeability, whether developmental or inflammatory driven, may facilitate brain exposure to MEVs, providing a pathway for maternal signals to shape neurodevelopment.

EVs are increasingly recognized as important mediators of intercellular communication within the central nervous system (CNS). For example, EVs derived from both the periphery and CNS (e.g., neurons, astrocytes) can interact with and regulate microglia function [25–27]. A recent in vitro study demonstrated that MEVs are readily taken up by homeostatic and polarized human microglia, influencing the abundance and enzymatic activity of a critical epigenetic modulator, DNA methyltransferase 1 [DNMT1; 27]. The study also illustrated a mechanistic association between DNMT1 regulation and MEV-miRNA 148a-5P.

miRNAs are small noncoding RNAs that regulate post-transcriptional gene expression and affect neural development, primarily by acting as repressors of target messenger RNA [28, 29]. Specific miRNA trafficked by MEVs are known to regulate synaptic development [see 15, 17, 30] and inflammatory responses, alongside other physiological functions [31]. Additionally, MEVs have beneficial effects in the gut through interactions with epithelial cells [14, 32] and may influence offspring health and development by directly influencing the microbiome via their miRNA cargos [6, 18, 32, 33]. These findings suggest that MEVs have multiple points of contact with CNS target sites by which they can modify offspring neurodevelopment through postnatal transcriptional signaling.

Maternal infections during pregnancy are relatively common [34] and have been linked to an increased risk of neurodevelopmental disorders in offspring [35]. To investigate the mechanisms underlying this association, animal models of maternal immune activation (MIA), where maternal immune stimulation during gestation induces neurodevelopmental impairments in offspring, are widely used and validated [36, 37]. While these gestational MIA models are supported by epidemiological evidence and offer strong translational relevance, comparatively little is known about the prevalence or risks of infections during the lactational period [38]. Recently, animal models of MIA during lactation have been evaluated [39–41], demonstrating that maternal immune challenges during the nursing period impact offspring neurodevelopmental outcomes similarly to the gestational models. Importantly, these behavioral effects occur despite minimal alterations in maternal care behaviors [39, 40, 42, 43]. This suggests that other variables, like milk-borne factors, may mediate these outcomes. While stressors experienced across lactation can influence maternal physiology and milk composition [10, 44], comprehensive analyses of traditional milk factors such as nutritional content, immunoglobulins, inflammatory cytokines, and even the maternal immunogen used to stimulate lactational MIA have not fully explained the effects on offspring neurodevelopment [39]. Therefore, alternative mechanisms must be considered.

Given the capacity of MEVs to deliver regulatory molecules to recipient cells and impart biological effects [6, 15, 16, 18], and their demonstrated involvement in the CNS [27], MEVs are a plausible candidate to mediate the effects of lactational MIA. Indeed, environmental experiences such as stress and illness modify the miRNA cargo of MEVs [7, 45–47], supporting the hypothesis that MEVs are sensitive to maternal environmental conditions and may relay this information to nursing offspring. Based on these findings, we hypothesize that lactational MIA alters the cargo of MEVs, leading to changes in the transcriptomic profile of the developing brain. These molecular changes may drive long-term alterations in gene expression, contributing to behavioral abnormalities observed in adulthood. Furthermore, because environmental enrichment (EE) has been shown to mitigate the behavioral consequences of gestational MIA [48–50] and to improve milk quality in healthy laboratory rats [51], we propose that an EE intervention may stabilize MEV cargos, protecting against the effects of lactational MIA. Understanding these mechanisms will provide insight into how the maternal environment shapes offspring neurodevelopment via milk-derived signaling pathways and may suggest strategies to enhance resilience in at-risk populations.

## Methods and experimental overview

**Fig. 1:**
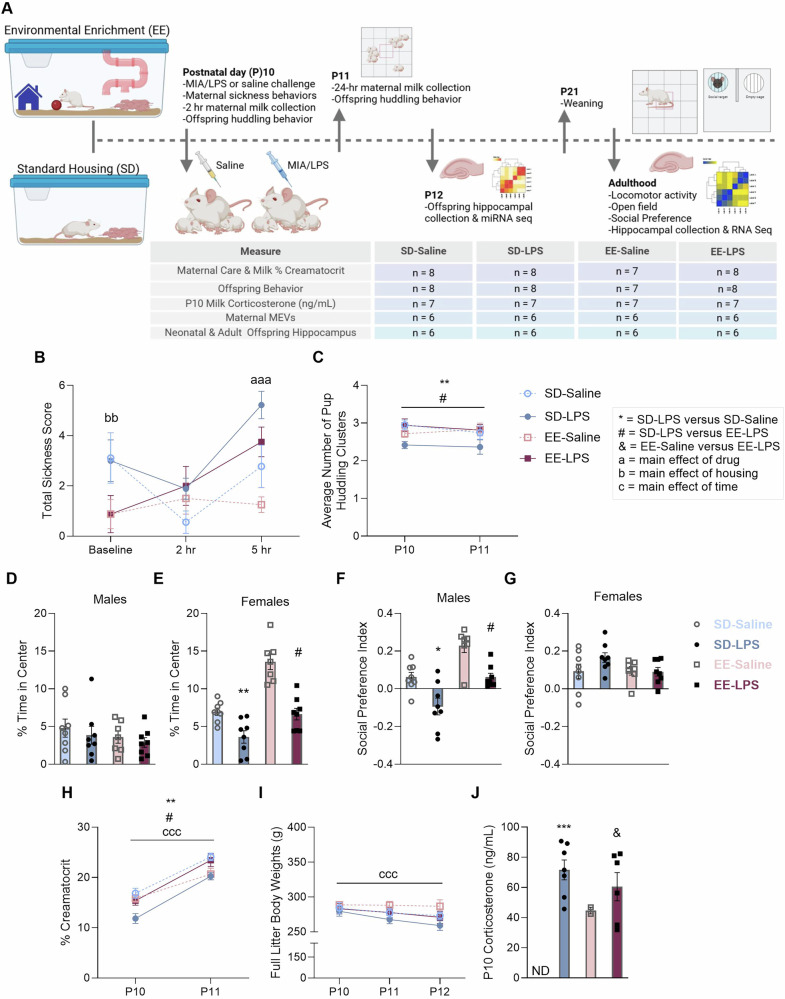
Environmental enrichment protects against lactational maternal immune activation (MIA) induced changes in offspring behavior. **A** Timeline of experimental procedures and group designations/sample sizes (n). **B** Maternal sickness behavior scores. **C** Neonatal huddling behavior (average number of pup clusters) across P10 and P11. Adult (**D**) male and (**E**) female percent (%) time in the center of the open field. Adult (**F**) male and (**G**) female social preference. **H** Percent (%) creamatocrit from P10-P11 and (**I**) the full litter body weights of nursing pups (g) across P10-P12. **J** Milk corticosterone (ng/mL) after MIA on P10; ND: non detectable values that fell below level of assay detection. Data are expressed as mean ± SEM; SD-Saline: n = 7–8; SD-LPS: n = 7–8; EE-Saline: n = 7; EE-LPS: n = 7–8. *p < 0.05, **p < 0.01, ***p < 0.001, SD-Saline versus SD-LPS; #p < 0.05, ##p < 0.01, ###p < 0.001, SD-LPS versus EE-LPS; ^&^p < 0.05, ^& &^p < 0.01, ^& & &^p < 0.001, EE-Saline versus EE-LPS; ^a^p < 0^.^05, ^aa^p < 0.01, ^aaa^p ^<^0.001, main effect of drug; ^b^p < 0.05, ^bb^p < 0.01, ^bbb^p < 0.001, main effect of housing; ^c^p < 0.05, ^cc^p < 0.01, ^ccc^p < 0.001, main effect of time; LPS lipopolysaccharide, P postnatal day. *Created in BioRender. Kentner, A. (2025)* https://BioRender.com/jrb10y6.

All animal protocols were approved by the MCPHS Institutional Animal Care and Use Committee and complied with AAALAC guidelines. The experimental timeline is illustrated in Fig. 1A. Complete methodological details are provided in the Supplementary Methods, Supplementary Table 1 [37], and DeRosa et al. [39, 51].

### Animals and housing

Sprague Dawley rats (Charles River, Wilmington) were bred and housed in either environmentally enriched (EE) or standard (SD) laboratory conditions as previously described [49, 51]. On postnatal day (P)10, lactating dams were administered either 100 µg/kg lipopolysaccharide (LPS; *Escherichia coli* O26:B6) or pyrogen-free saline via intraperitoneal injection (n = 7–8 litters/group). Dams were separated from their litters to allow milk to accumulate for 2 h post-injection while litters were kept warm on a heating pad [39, 51]. Based on previous work, this period of separation does not induce a strong corticosterone response in neonates [52].

### Maternal assessments

Sickness behavior was evaluated at baseline, 120-, and 300 min post-injection using a composite score of ptosis, piloerection, and lethargy (0 = none, 1 = mild, 2 = severe), by blinded investigators, as adapted from prior studies [48, 53, 54]. Maternal care was observed on P9–P11 during AM and PM sessions. Behavioral metrics included pup licking/grooming frequency and time spent on the nest across six 1 min intervals [39, 51].

### Milk collection and analysis

Two hours after injection, dams were anesthetized with isoflurane and administered oxytocin (0.2 mL; 20 USP/mL, i.p.) to induce milk letdown [39, 51]; based on their pharmacokinetic profiles, these drugs are not absorbed by offspring [55–57]. Approximately 2.0 mL of milk was manually expressed and collected over 50–60 min. Dams were returned to their litters once recovered from anesthesia. Milk was collected again 24 h later on P11. Percent creamatocrit was determined following procedures previously outlined [39, 51, 58], and 100 µL of milk was stored at –80 °C for corticosterone assays. Samples were thawed and rotated overnight, then analyzed using a small sample ELISA protocol (#ADI-900–097, Enzo Life Sciences; 1:40 dilution) [39, 51].

### Milk-derived extracellular vesicles (MEVs)

**Fig. 2:**
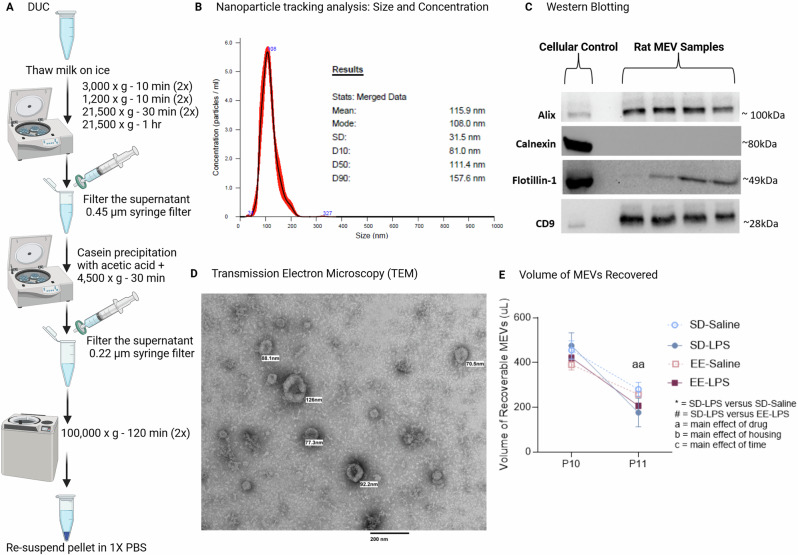
Characterization of milk-derived extracellular vesicles (MEVs) isolated from rat milk following maternal immune activation (MIA). **A** The differential ultracentrifugation (DUC) and serial filtration protocol followed for MEV isolation. **B** Size (nm) and concentration (particles/mL) of MEVs as determined by nanoparticle tracking analysis (NTA) with a mean size of 115.9 ± 31.5 nm. **C** The presence or absence of positive (Alix, Flotillin-1, CD9; cellular control: human MEVs) and negative (Calnexin; cellular control: Hep G2 cells) protein markers for MEVs as determined by western immunoblotting. **D** Transmission electron microscopy (TEM) of a representative MEV pellet. Scale bar is 200 nm with some individual MEVs identified in sizes ranging from 70.5–126 nm. **E** Total yield of MEVs recovered. Data are expressed as mean ± SEM, n = 6. ^aa^p < 0.01, main effect of drug; LPS lipopolysaccharide, P postnatal day; SD: standard housing. *Created in BioRender. Kentner, A. (2025)* https://BioRender.com/x9rk66v.

Following centrifugation to remove the cream layer, MEVs were isolated from the supernatant using differential ultracentrifugation and filtration (DUC), as previously outlined [2, 3, 27, 59] (Fig. 2A) and compliant with MISEV 2023 guidelines [60]. Casein was precipitated [61], and whey fractions were ultracentrifuged to pellet MEVs, which were washed and resuspended in PBS. MEVs were characterized per MISEV 2023 guidelines using nanoparticle tracking analysis (NTA), western blotting, and transmission electron microscopy (TEM). NTA determined MEV size and concentration (Fig. 2B). Western immunoblotting confirmed the presence of markers CD-9, Alix, Flotillin-1, and the absence of Calnexin (Fig. 2C); these protein markers were assessed qualitatively to verify MEV identity [27, 59, 60]. TEM verified MEV morphology (Fig. 2D).

### Offspring assessments

The offspring of saline and MIA dams were evaluated on neonatal huddling behavior (a proxy for neonatal thermoregulation), two hours after reuniting with their dams on P10 and P11 [39, 62]. Litters were weaned into same-sex pairs on P21, maintaining their original housing assignments. Starting on P70, one male and one female offspring from each litter were assessed on the open field and social preference tests (n = 7–8 litters/group/sex; Groups: SD-Saline: n = 8, SD-LPS: n = 8, EE-Saline: n = 7, EE-LPS: n = 8, collected across two cohorts) [39, 49, 63]. One male and one female from each litter was euthanized with isoflurane, and whole (at P12) or ventral [64–66] (at P74) hippocampus dissected, frozen on dry ice and stored at −80 °C. Sample sizes were determined using 80% as a desired power to observe a significant difference (α level of 0.05), and an effect size of 0.25.

### RNA Extraction and Sequencing

Total RNA was extracted from hippocampal tissues using the RNeasy Lipid Tissue Mini Kit (74804, Qiagen) and from MEV samples using the Exosomal RNA Isolation Kit (58000, Norogen Biotek), according to the manufacturer’s instructions. Samples were quantified using Qubit 2.0 Fluorometer (ThermoFisher Scientific) and RNA integrity was checked with 4200 TapeStation (Agilent Technologies).

#### MEV small RNA-seq (n = 6 litters/group)

Libraries were prepared using NEB Small RNA Library Prep Kit (New England Biolabs), validated on TapeStation, quantified using Qubit 2.0 Fluorometer, sequenced on Illumina NovaSeq (2x150bp PE), and de-multiplexed using Illumina’s bcl2fastq 2.20 software.

#### Neonatal hippocampus small RNA-seq (n = 6 litters/group/sex)

Libraries were prepared using Illumina TruSeq Small RNA Library Prep Kit, cDNA constructs purified via BluePippin, validated on TapeStation, quantified using Qubit 2.0 Fluorometer, and sequenced on Illumina NextSeq 2000 (1x50bp).

#### Adult hippocampus RNA-seq (n = 6 litters/group/sex)

Strand-specific libraries were generated using NEBNext Ultra II Directional RNA Library Prep Kit (Illumina), validated on TapeStation, quantified using Qubit 2.0 Fluorometer, sequenced on Illumina NovaSeq (2x150bp PE), and de-multiplexed using Illumina’s bcl2fastq 2.20 software.

### Sequencing analysis

Differentially expressed miRNAs and genes were identified based on a p < 0.01, (FC) > 2.0. Heatmaps were generated using Multiple Experiment Viewer. Venn diagrams were generated using DeepVenn (https://www.deepvenn.com). Gene ontology was determined using the DAVID functional annotation cluster tool (https://david.ncifcrf.gov/), while miRNA Enrichment Analysis and Annotation Tool (miEAA) was used to functionally annotate the different sets of miRNAs. Bubble plots and pie charts were generated in GraphPad Prism 10.4.2.

### Statistical analyses

Data were analyzed using Prism (GraphPad) or SPSS (IBM). ANOVAs (Housing x MIA x Time or Housing x MIA) were used to evaluate milk % creamatocrit and behavioral endpoints, unless there were violations to the assumption of normality (Shapiro-Wilk test) in which case Kruskal-Wallis tests were employed (expressed as *X*^2^). To assess differences in the proportion of samples with detectable P10 milk corticosterone concentrations, the Fisher-Freeman-Halton exact test [67] was used to appropriately account for the undetectable hormone levels in the SD-Saline group. Partial eta-squared (ηp^2^) is reported as an index of effect size for the ANOVAs [68]. Both male and female animals were included, and separate analyses were run for each sex [39, 51, 68, 69, 70]. All data are expressed as mean ± SEM.

## Results

### EE protected against lactational MIA-induced changes in offspring behavior

Maternal immune challenge during lactation significantly increased sickness behaviors in dams, regardless of housing assignment, validating the model. A MIA by time interaction identified an increased severity of sickness behavior in LPS treated dams 5 h post immune challenge, compared to saline dams (Fig. 1B). A housing by time interaction indicated that SD dams appeared less healthy than EE dams at baseline, consistent with prior findings [51]. SD dams, confined to smaller cages, nurse more due to a limited ability to escape from pups. SD housing is associated with displays of “pressing” behavior where dams press their ventral side against the cage, seemingly to hide their teats and take a break from the energy-intensive nursing task [51].

Huddling behavior was significantly reduced in SD-LPS neonatal offspring compared to neonates from SD-Saline dams (MIA by housing interaction; average number of clusters Fig. 1C). Diminished neonatal huddling behavior was buffered by EE (SD-LPS versus EE-LPS).

In the open field test, MIA by housing interactions were observed for both adult male and female offspring in terms of distance traveled (Supplementary Fig. 1A, B). However, follow up tests were not significant in males; female EE-LPS rats traveled less than SD-LPS females. While males were not affected, SD-LPS female offspring spent a reduced percentage of time in the center of an open field compared to SD-Saline females (MIA by housing interaction: Fig. 1D, E). This MIA induced avoidance behavior was prevented by EE housing. In contrast, male SD-LPS adult offspring had a decreased social preference index compared to SD-Saline males (MIA by housing interaction: Fig. 1F, G). Complex EE housing also protected against the MIA associated alteration in male social behavior.

Maternal care differences do not appear to account for these effects since there were no significant group differences in terms of total time on nest or the number of pup-directed licking or grooming bouts (Supplementary Fig. 1C, D). While previous work has shown housing differences in the maternal care of untreated rats [51], stress induced by the LPS/Saline injections may have normalized the amount of maternal care displayed across SD and EE groups in the present study. Statistics reported in Supplemental Table 3.

### Housing condition regulates lactational MIA-induced changes in MEV-miRNA cargos

Building on our previous observations that MIA reduces milk quality [39] while EE improves it [51], we investigated whether EE housing could mitigate MIA-induced changes to milk composition. Percent creamatocrit was decreased in the milk of SD-LPS dams compared to SD-Saline, which was mitigated by EE housing (MIA x Housing interaction; main effect of time; Fig. 1H). However, the body weights of nursing pups were not affected by either MIA or housing across P10-P12 (p > 0.05; Fig. 1I) nor were they different at weaning (p > 0.05; Supplementary Fig. 1E). Milk corticosterone, known to influence offspring development and temperament [71, 72], differed significantly across the experimental groups (p = 0.001). Specifically, milk corticosterone was detectable following MIA on P10 (SD-Saline versus SD-LPS: p = 0.001; EE-Saline versus EE-LPS: p = 0.015) which was not reduced by EE (SD-LPS versus EE-LPS: p > 0.05; Fig. 1J). Therefore, it is unlikely that attenuation of corticosterone underlies the protective effects of EE on behavior. The full statistical results are reported in Supplemental Table 3.

**Fig. 3:**
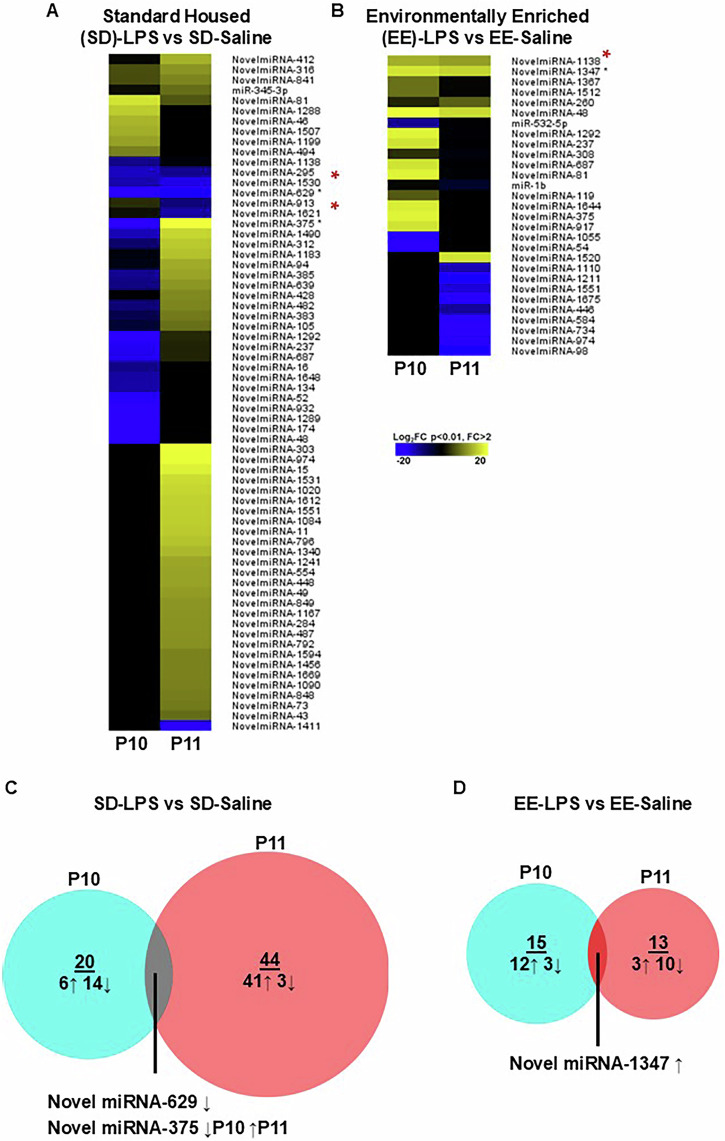
Lactational maternal immune activation (MIA) alters milk-derived extracellular vesicles (MEV) miRNA cargos, which are buffered by environmental enrichment (EE) housing. miRNA clustered in the heatmaps display the Log_2_FC p < 0.01, FC > 2 in (**A**) SD-LPS versus SD-Saline and (**B**) EE-LPS versus EE-Saline MEVs at P10 and P11. Venn diagrams show the number of differentially expressed miRNA in P10 (blue) and P11 (red), and their overlapping miRNAs and direction of change ( ↑ upregulated, ↓ downregulated) for (**C**) SD-LPS versus SD-Saline and (**D**) EE-LPS versus EE-Saline. LPS lipopolysaccharide, P postnatal day; SD: standard housing; n = 6. *If significant at P10 and P11.

Although the total yield of MEVs recovered during the isolation process was reduced by MIA on P11 (MIA x Time interaction; Fig. 2E) this was also not protected by EE in this model. Given the characteristics of MEVs to carry bioactive cargos such as miRNAs [15], we turned our interests towards the effects of MIA and EE on MEV cargo composition. The cargo of MEVs isolated from SD-LPS dams showed 66 differentially expressed miRNAs compared to MEVs from SD-Saline dams. Specifically, 22 miRNAs were differentially expressed at P10, while 46 miRNAs were differentially expressed at P11, indicating that it takes about 24 h for peak MEV-miRNA changes to appear (Fig. 3A, C). Curiously, in the cargo of MEVs isolated from EE-LPS dams only 29 miRNAs, of which 16 at P10 and 14 at P11, were differentially expressed compared to MEVs from EE-Saline dams (Fig. 3B, D). This means that, when dams were raised in EE, the number of LPS-induced miRNAs did not vary the day after the LPS challenge (P11) as opposed to what was observed in dams raised in SD (Supplementary Fig. 2). In addition, we found that novel miRNA-629 was downregulated both at P10 and P11 in SD-LPS MEVs compared to SD-Saline MEVs, while the regulation of novel miRNA-375 diverged between P10 and P11. Finally, novel miRNA-1347 was upregulated in the MEVs of LPS-treated dams compared to Saline-treated dams raised in EE, regardless of timepoint (Fig. 3C, D). Together, these data highlight the protective effects of EE on MEV-miRNAs both acutely and chronically, given the sustained ability of enrichment to regulate this cargo.

### The composition of miRNAs in the nursing offspring hippocampus correlates with MEV-miRNA cargo as a function of housing

**Fig. 4:**
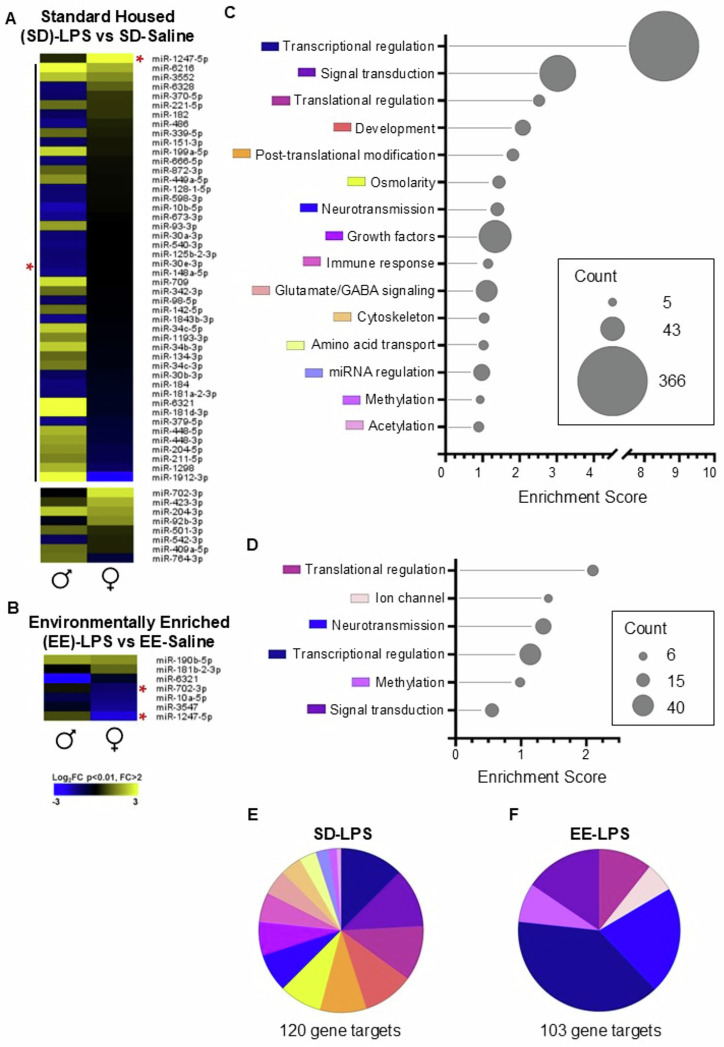
Lactational maternal immune activation (MIA) alters miRNA expression in the hippocampus of nursing offspring, which is buffered by environmental enrichment (EE) housing. miRNA clustered in the heatmaps display the Log_2_FC p < 0.01, FC > 2 in (**A**) SD-LPS versus SD-Saline and (**B**) EE-LPS versus EE-Saline neonatal hippocampus on P12 for male and female offspring. (**C**, **D**) The bubble plots show the enrichment score of gene pathways generated using DAVID by clustering genes predicted as miRNA-mRNA targets (mirdb.org, target score >80). Gene counts per cluster are represented as bubble size. Gene pathways highlighted are top unique clusters from (**C**) Figure A, and (**D**) Figure B. (**E**, **F**) Pie charts to visualize gene count distribution of pathways highlighted in (**E**) Figure C and (**F**) Figure D. LPS lipopolysaccharide, P postnatal day, SD standard housing, n = 6; *if FDR < 0.05. Edited in BioRender. Kentner, A. (2025) https://BioRender.com/w5acrdr.

We then sought to explore the expression of miRNAs in the hippocampus of the nursing offspring (P12) of dams whose MEV-miRNA cargo was significantly affected by MIA as a function of EE. LPS caused differential expression of 50 miRNAs (45 miRNAs, adj p-value < 0.05) in SD males and 4 miRNAs (miR-1247-5p, adj p-value < 0.05) in SD females (Fig. 4A). In contrast, as also observed in MEVs from EE dams, EE dramatically reduced the number of differentially expressed LPS-related miRNAs in the hippocampus compared to miRNAs induced by LPS in SD rats. Namely, LPS regulated 2 miRNAs in EE males and 5 miRNAs in EE females (2 miRNAs, adj p-value < 0.05) compared to their respective Saline-treated EE controls (Fig. 4B).

We were interested in understanding the genomic function of differentially expressed miRNAs regulated by LPS as a function of housing across groups. To predict the miRNA-mRNA targets, we used miRDB (threshold target score >80) combined with DAVID to cluster predicted genes according to their gene ontology. In selected pathways of interest, we found that miRNA-targeted genes in LPS rats (120 in SD rats and 103 in EE rats) were involved in several cellular functions including transcriptional and translational regulation, signal transduction, neurotransmission, as well as epigenetic regulation, regardless of their housing condition. Notably, glutamatergic/GABAergic signaling, immune response, growth factors, and development-related pathways were targeted in LPS rats raised in SD, but not in EE (Fig. 4C-F).

Building on the evidence that LPS caused behavioral alterations in adulthood that depended on early housing condition, we investigated the genomic profile in the ventral hippocampus of the adult offspring. The downregulation of *Thbs1*, a gene involved in autism spectrum disorder risk [73, 74], in the hippocampus of male rats whose mothers were raised in SD and challenged with LPS, was consistent with the upregulation of the miRNA regulator, miR-709, in the hippocampus at P12. The same was true for the upregulation of the gene *Naa11*, also involved in autism susceptibility [75], whose regulator, miR-128-1-5p, was downregulated at P12 in the hippocampus of male rats whose mothers were raised in SD and challenged with LPS. We found that miRNA targets at P12 predicted the regulation of several genes that belonged to the same gene superfamily of the ones that were differentially expressed in the adult ventral hippocampus (Supplemental Table 2).

**Fig. 5:**
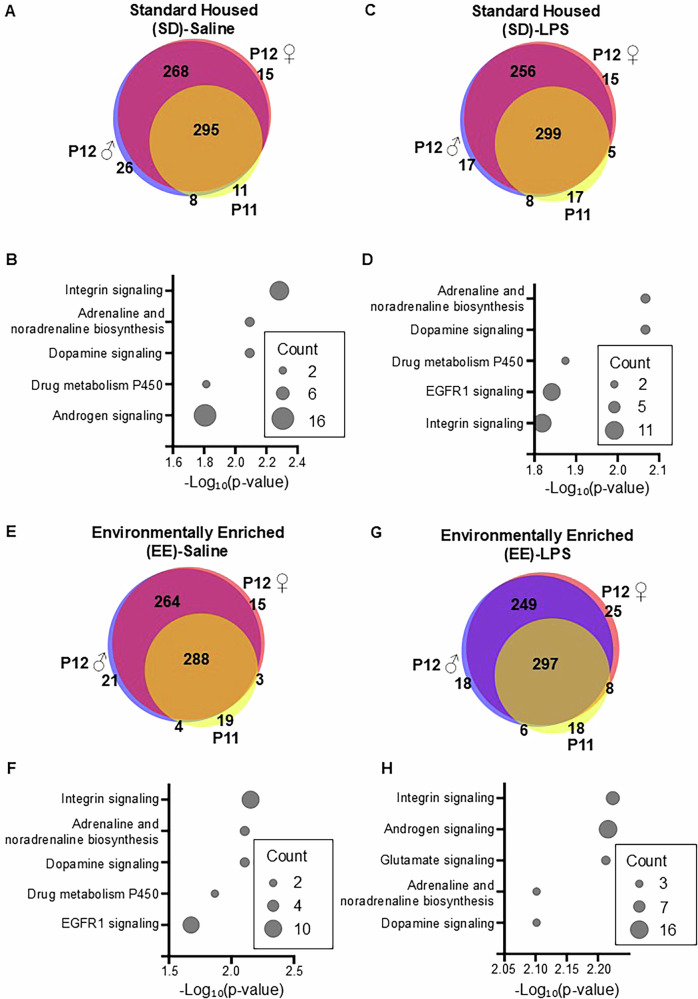
Overlapping miRNAs matched between maternal milk-derived extracellular vesicles (MEVs) and the hippocampus of their nursing male and female neonatal offspring. **A**, **C**, **E**, **G** Venn diagrams showing the number of miRNAs found in MEVs at P11 (yellow) and the hippocampus of male (blue) and female (red) offspring at P12, and their overlapping miRNA in groups (**A**) SD-Saline, (**C**) SD-LPS, (**E**) EE-Saline, and (**G**) EE-LPS. **B**, **D**, **F**, **H** The bubble plots show the -Log₁₀ (p-value) of the top 5 enriched miRWalk pathways identified for the overlapping miRNA in (**B**) Figure A, (**D**) Figure C, (**F**) Figure E, and (**H**) Figure G, using the miRNA Enrichment Analysis and Annotation Tool (ccb-compute2.cs.uni-saarland.de/mieaa). miRNA count per pathway is indicated by bubble size. LPS lipopolysaccharide, P postnatal day, SD standard housing, EE environmental enrichment; n = 6.

To assess whether miRNAs differentially expressed in MEVs were also affected in the hippocampus of the nursing offspring at P12, we compared the miRNA counts in both miRNA-seq datasets. While the overall pattern of common expression between MEV and hippocampus did not depend on treatment, housing, or sex, there were differences in the genomic function of miRNA sets across groups. Using miRNA Enrichment Analysis and Annotation Tool, we found that, out of the top five pathways, integrin and dopamine signaling, as well as adrenalin and noradrenalin biosynthesis, were regulated across all experimental groups (Fig. 5A-H). However, the regulation of the glutamate signaling pathway was exclusive to LPS-treated groups raised in EE (Fig. 5G, H). Additionally, the drug metabolism P450 pathway was regulated in all groups except EE-LPS. Again, this suggests that EE has unique effects on LPS-induced transcriptional regulation.

## Discussion

**Fig. 6:**
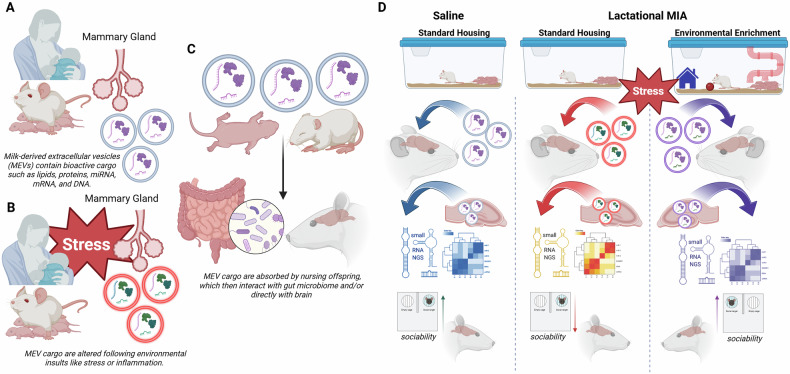
Overarching hypothesis of milk-derived extracellular vesicles (MEVs) as a mechanism of postnatal neuroimmune programming. **A** Nursing mammals pass MEVs through their milk to their feeding offspring. **B** Insults such as inflammatory stressors change the composition of MEV cargo. **C** Data suggest that when absorbed by nursing offspring, MEVs can interact with the gut microbiome and/or cross into and accumulate in the offspring brain. **D** We propose that maternal immune activation (MIA) alters the composition of MEV cargo, which then infiltrates the feeding offspring. Through either direct (brain) or indirect (e.g., gut microbiome) interactions, the altered MEV cargo (e.g., miRNAs), lead to transcriptional changes in brain regions like the hippocampus, affecting later life behavior in offspring. Environmental enrichment interventions can neutralize these changes, conserving behavior. Created in BioRender. Kentner, A. (2025) https://BioRender.com/50zpt07.

Postpartum infections are known to affect breastfeeding people, however the consequences of illness on lactation and associated offspring outcomes are understudied [38]. Despite awareness of the importance of maternal health in the postpartum period, interventions aimed at supporting caregivers are often underutilized and undervalued. Compounding this gap is a limited understanding of the biological mechanisms through which such interventions may exert their effects. In this context, we identify MEV-miRNAs as a dynamic signaling pathway capable of conveying both stress-induced and protective environmental cues, from mothers to their offspring, during the lactational period. This highlights a potential mechanistic link between maternal experience and early developmental programing (see Fig. 6 for summary of proposed mechanisms).

Consistent with prior work, we found that MIA increased corticosterone levels in maternal milk [39], a glucocorticoid known to influence stress responsivity and emotional regulation in offspring [72, 76]. However, EE housing did not attenuate MIA-induced elevations in milk corticosterone. This suggests that corticosterone is unlikely to mediate the protective effects of EE observed in this lactational MIA model. Moreover, while MIA caused a reduction in creamatocrit that was rescued by EE, offspring body weights were unaffected during the neonatal and weaning periods. Adult EE males did weigh more than their adult SD counterparts; however, the absence of the early weight differences, in combination with the early transcriptional and behavioral changes, supports the idea that other milk constituents may play a more prominent role in developmental programming.

Instead, our findings point to MEVs and their miRNA cargos as key mediators of environmentally driven signaling between mothers and nursing offspring. Here, MIA induced robust alterations in MEV miRNA cargo, particularly in mothers housed under standard laboratory conditions. In contrast, dams reared in EE displayed fewer MIA-induced changes in MEV-miRNA profiles, supporting the notion that enrichment stabilizes maternal biological outputs under stress. Notably, in MEVs from standard housed dams, novel miRNA-629 was consistently downregulated by MIA across both sampling days (P10 and P11), while novel miRNA-375 showed a biphasic expression pattern. These miRNAs predicted by miRDeep 2.0, and clustered by DAVID, targeted genes involved in transcriptional regulation, ion channel function, and signal transduction, pathways critical to neurodevelopment and synaptic plasticity [77, 78]. In MEVs from enriched dams, MIA upregulated novel miRNA-1347, which is predicted to target the expression of Tbl1xr1, a gene implicated in developmental disorders whose regulation confers protection [79–82]. This suggests that EE-modified MEV cargo programs developmental adaptation in the offspring via transcriptional processes. Importantly, the influence of the environment on MEVs is not transient.

The regulatory potential of MEV-miRNAs was consistent with changes observed in the neonatal hippocampus on P12. Indeed, LPS challenge resulted in a significant *downregulation* of miRNAs in the MEVs from SD mothers on P11, suggesting that there were fewer regulating mechanisms following MIA [28, 29]. While it is also important to keep in mind the role of other MEV cargos (e.g., lipid and protein content), of the significant MEV-miRNAs in the EE housed dams there was more *upregulation*, suggesting that there may have been more regulatory mechanisms at play. Similar to the patterns of MIA-induced differentially expressed miRNAs in the MEVs, enrichment reduced the number of differentially expressed hippocampal miRNAs, indicating that EE can buffer against stress-induced transcriptomic modifications. Together, this suggests that EE housing stabilized the offspring brain during MIA through MEV delivery. Several miRNAs were expressed in both MEVs and the neonatal hippocampus, suggesting potential transfer of regulatory signals from milk to brain. Moreover, miR-709 and miR-128-1-5p were differentially expressed in neonatal hippocampus and showed the expected directional changes in their predicted gene targets (*Thbs1* and *Naa11*) in the adult hippocampal transcriptome. Cross-tissue miRNA-mRNA targets provide compelling support to the hypothesis that MEVs are sensitive to maternal environmental conditions and may relay this information to the developing offspring brain [7].

Lactational MIA induced behavioral alterations in offspring that were also buffered by enrichment. In female offspring, MIA reduced the percent of time animals explored the center of the open field arena, indicative of heightened anxiety-like behavior; in male offspring, social preference was diminished by the MIA challenge. Importantly, EE exposure prevented these outcomes in both sexes, consistent with prior studies demonstrating the protective effects of enriched housing on offspring stress regulation and social engagement [48–50, 83]. Interestingly, differences in maternal care were not observed across groups, suggesting that EE protection was not mediated by changes in overt maternal behaviors, such as time spent on the nest or frequency of licking/grooming. This is noteworthy, as enriched housing can modulate maternal care under non-stress conditions [51], but the added “injection stress” from administering either LPS or saline during lactation may have masked group differences. Maternal care is sensitive to laboratory manipulations, and these findings caution against the assumption that vehicle injections represent a neutral control. It is possible that relatively higher levels of parental support or enrichment may be needed, even in higher resource settings, to buffer against acute stressors. In general, it appears that the behavioral buffering effects of EE may instead involve more subtle or molecular-level changes in maternal signaling via MEVs in this lactational MIA model, rather than direct maternal care interactions.

In addition to sex-specific behavioral phenotypes, following lactational MIA, there were significantly different miRNA profiles in male and female hippocampus. The differential expression of MIA-induced hippocampal miRNAs in SD offspring was dramatically greater in males than in females, aligning with previous work showing sex-specific vulnerability to early life stressors [84]. While SD-LPS females had fewer changes in miRNA expression, it was notable that miR-1247-5p was differentially expressed in the hippocampus of this group. In vivo studies have identified this novel miRNA as neuroprotective following injury, reporting that it affected developmentally regulated biological processes such as differentiation, transcription regulation, and mitochondrial function [85]. This raises the possibility that females possess unique compensatory mechanisms that are activated under stress.

We acknowledge that there are limitations one should consider when interpreting some of the findings. First, the dynamic nature of milk composition across the lactational period limits the temporal generalizability of our results. Our study only sampled across two days, and it remains unclear whether certain phases of lactation may be more or less responsive to either MIA or EE. Second, although we identified associations between miRNAs in MEVs and neonatal hippocampus, direct mechanistic evidence for MEV transfer and functional integration in the offspring brain is lacking. It is not known whether neonatal hippocampal miRNA profiles were directly reflective of deposited maternal MEV cargo or rather due to indirect actions between MEVs and the microbiome, influencing the hippocampal transcriptome, for example. Future studies using fluorescently labeled MEVs and individual miRNA cargo, in addition to MEV depletion and supplementation approaches are needed to directly test the uptake and influence of MEVs in brain. MEVs themselves carry multiple types of cargo, including lipids and peptides that can influence neurodevelopment, in addition to the miRNAs evaluated here. Finally, while our study highlights MEVs as plausible mediators of environmental signaling, we do not exclude interactive effects with other nutritive and non-nutritive milk constituents such as lipids, peptides, and glucocorticoids [39], or factors such as pup separation which could have contributed to MEV-miRNA programming. The complexity of milk composition argues against a single-pathway explanation, and it is likely that additive or synergistic interactions across multiple milk and environmental factors are shaping neurodevelopmental trajectories.

## Conclusions

Breastfeeding remains the recommended source of nutrition during most maternal illnesses, as many pathogens including influenza, COVID-19, and foodborne agents are not transmitted directly through breast milk, yet antibodies against these pathogens are transmitted to offspring [86]. Critically, maternal illness and stress can alter milk composition, including the regulatory cargo of MEVs. Our findings demonstrate that MEV-miRNAs are sensitive to the maternal environment and can shape offspring neurodevelopment, highlighting the need for increased support for breastfeeding individuals. This includes a need for improved education and communication pathways to ensure breastfeeding individuals are informed and supported [87]. Indeed, enrichment exposure stabilized MEV cargo and mitigated the effects of MIA on offspring, positioning MEV-miRNAs as dynamic programming signals by which both stress-induced and protective cues are relayed. Given evidence linking breastfeeding to reduced neurodevelopmental disorder risk [88], our study positions MEVs as a key mechanism by which maternal experiences influence infant brain development, with important implications for maternal-infant health policy and care.

## Supplementary information

Supplemental Methods and Experimental Overview

Supplemental Table 1.

Supplemental Figure 1

Supplemental Figure 2

Supplemental Table 2.

Supplemental Table 3.

## Data Availability

Sequencing datasets have been deposited to GEO (GSE300302, GSE300818). All other data is presented in the article files or available upon request.

## References

[1] Aplin JD, Myers JE, Timms K, Westwood M. Tracking placental development in health and disease. Nat Rev Endocrinol. 2020;16:479–94. 10.1038/s41574-020-0372-6. 32601352 10.1038/s41574-020-0372-6

[2] Morgan CP, Chan JC, Bale TL. Driving the next generation: paternal lifetime experiences transmitted via extracellular vesicles and their small RNA cargo. Biol Psychiatry. 2019;85:164–71. 10.1016/j.biopsych.2018.09.007. 30580777 10.1016/j.biopsych.2018.09.007 PMC6309802

[3] Chan JC, Morgan CP, Adrian Leu N, Shetty A, Cisse YM, Nugent BM, et al. Reproductive tract extracellular vesicles are sufficient to transmit intergenerational stress and program neurodevelopment. Nat Commun. 2020;11:1499. 10.1038/s41467-020-15305-w. 32198406 10.1038/s41467-020-15305-w PMC7083921

[4] Sánchez-Rubio M, Abarzúa-Catalán L, Del Valle A, Méndez-Ruette M, Salazar N, Sigala J, et al. Maternal stress during pregnancy alters circulating small extracellular vesicles and enhances their targeting to the placenta and fetus. Biol Res. 2024;57:70. 10.1186/s40659-024-00548-4. 39342314 10.1186/s40659-024-00548-4 PMC11438166

[5] Zhou F, Ebea P, Mutai E, Wang H, Sukreet S, Navazesh S, et al. Small extracellular vesicles in milk cross the blood-brain barrier in murine cerebral cortex endothelial cells and promote dendritic complexity in the hippocampus and brain function in C57BL/6J mice. Front Nutr. 2022;9:838543. 10.3389/fnut.2022.838543. 35600828 10.3389/fnut.2022.838543 PMC9121399

[6] Zempleni J, Sukreet S, Zhou F, Wu D, Mutai E. Milk-derived exosomes and metabolic regulation. Annu Rev Anim Biosci. 2019;7:245–62. 10.1146/annurev-animal-020518-115300. 30285461 10.1146/annurev-animal-020518-115300

[7] Bozack AK, Colicino E, Rodosthenous R, Bloomquist TR, Baccarelli AA, Wright RO, et al. Associations between maternal lifetime stressors and negative events in pregnancy and breast milk-derived extracellular vesicle microRNAs in the programming of intergenerational stress mechanisms (PRISM) pregnancy cohort. Epigenetics. 2021;16:389–404. 10.1080/15592294.2020.1805677. 32777999 10.1080/15592294.2020.1805677 PMC7996083

[8] Yáñez-Mó M, Siljander PR, Andreu Z, Zavec AB, Borràs FE, Buzas EI, et al. Biological properties of extracellular vesicles and their physiological functions. J Extracell Vesicles. 2015;4:27066. 10.3402/jev.v4.27066. 25979354 10.3402/jev.v4.27066 PMC4433489

[9] EL Andaloussi S, Mäger I, Breakefield XO, Wood MJ. Extracellular vesicles: biology and emerging therapeutic opportunities. Nat Rev Drug Discov. 2013;12:347–57. 10.1038/nrd3978. 23584393 10.1038/nrd3978

[10] Wijenayake S, Martz J, Lapp HE, Storm JA, Champagne FA, Kentner AC. The contributions of parental lactation on offspring development: It’s not udder nonsense! Horm Behav. 2023;153:105375. 10.1016/j.yhbeh.2023.105375. 37269591 10.1016/j.yhbeh.2023.105375 PMC10351876

[11] Ames SR, Lotoski LC, Azad MB. Comparing early life nutritional sources and human milk feeding practices: personalized and dynamic nutrition supports infant gut microbiome development and immune system maturation. Gut Microbes. 2023;15:2190305. 10.1080/19490976.2023.2190305. 37055920 10.1080/19490976.2023.2190305 PMC10114993

[12] de Weerth C, Aatsinki AK, Azad MB, Bartol FF, Bode L, Collado MC, et al. Human milk: From complex tailored nutrition to bioactive impact on child cognition and behavior. Crit Rev Food Sci Nutr. 2023;63:7945–82. 10.1080/10408398.2022.2053058. 35352583 10.1080/10408398.2022.2053058

[13] Victora CG, Bahl R, Barros AJ, França GV, Horton S, Krasevec J, et al. Breastfeeding in the 21st century: epidemiology, mechanisms, and lifelong effect. Lancet. 2016;387:475–90. 10.1016/S0140-6736(15)01024-7. 26869575 10.1016/S0140-6736(15)01024-7

[14] Hock A, Miyake H, Li B, Lee C, Ermini L, Koike Y, et al. Breast milk-derived exosomes promote intestinal epithelial cell growth. J Pediatr Surg. 2017;52:755–9. 10.1016/j.jpedsurg.2017.01.032. 28188035 10.1016/j.jpedsurg.2017.01.032

[15] Jiang X, You L, Zhang Z, Cui X, Zhong H, Sun X, et al. Biological properties of milk-derived extracellular vesicles and their physiological functions in infant. Front Cell Dev Biol. 2021;9:693534. 10.3389/fcell.2021.693534. 34249944 10.3389/fcell.2021.693534 PMC8267587

[16] Khanam A, Ngu A, Zempleni J. Bioavailability of orally administered small extracellular vesicles from bovine milk in C57BL/6J mice. Int J Pharm. 2023;639:122974. 10.1016/j.ijpharm.2023.122974. 37105241 10.1016/j.ijpharm.2023.122974 PMC10175213

[17] Lönnerdal B. Human milk MicroRNAs/exosomes: composition and biological effects. Nestle Nutr Inst Workshop Ser. 2019;90:83–92. 10.1159/000490297. 30865991 10.1159/000490297

[18] Melnik BC, Stremmel W, Weiskirchen R, John SM, Schmitz G. Exosome-derived MicroRNAs of human milk and their effects on infant health and development. Biomolecules. 2021;11:851. 10.3390/biom11060851. 34200323 10.3390/biom11060851 PMC8228670

[19] Manca S, Upadhyaya B, Mutai E, Desaulniers AT, Cederberg RA, White BR, et al. Milk exosomes are bioavailable and distinct microRNA cargos have unique tissue distribution patterns. Sci Rep. 2018;8:11321. 10.1038/s41598-018-29780-1. 30054561 10.1038/s41598-018-29780-1 PMC6063888

[20] Chen CC, Liu L, Ma F, Wong CW, Guo XE, Chacko JV, et al. Elucidation of exosome migration across the blood-brain barrier model in vitro. Cell Mol Bioeng. 2016;9:509–29. 10.1007/s12195-016-0458-3. 28392840 10.1007/s12195-016-0458-3 PMC5382965

[21] Mallard C, Ek CJ, Vexler ZS. The myth of the immature barrier systems in the developing brain: role in perinatal brain injury. J Physiol. 2018;596:5655–64. 10.1113/JP274938. 29528501 10.1113/JP274938 PMC6265562

[22] Saunders NR, Habgood MD, Dziegielewska KM. Barrier mechanisms in the brain, II. immature brain. Clin Exp Pharmacol Physiol. 1999;26:85–91. 10.1046/j.1440-1681.1999.02987.x. 10065326 10.1046/j.1440-1681.1999.02987.x

[23] Stolp H, Neuhaus A, Sundramoorthi R, Molnár Z. The long and the short of it: gene and environment interactions during early cortical development and consequences for long-term neurological disease. Front Psychiatry. 2012;3:50. 10.3389/fpsyt.2012.00050. 22701439 10.3389/fpsyt.2012.00050 PMC3372875

[24] Haney MJ, Klyachko NL, Harrison EB, Zhao Y, Kabanov AV, Batrakova EV. TPP1 delivery to lysosomes with extracellular vesicles and their enhanced brain distribution in the animal model of batten disease. Adv Healthc Mater. 2019;8:e1801271. 10.1002/adhm.201801271. 30997751 10.1002/adhm.201801271 PMC6584948

[25] Li Y, Yang YY, Ren JL, Xu F, Chen FM, Li A. Exosomes secreted by stem cells from human exfoliated deciduous teeth contribute to functional recovery after traumatic brain injury by shifting microglia M1/M2 polarization in rats. Stem Cell Res Ther. 2017;8:198. 10.1186/s13287-017-0648-5. 28962585 10.1186/s13287-017-0648-5 PMC5622448

[26] Paolicelli RC, Bergamini G, Rajendran L. Cell-to-cell communication by extracellular vesicles: focus on microglia. Neuroscience. 2019;405:148–57. 10.1016/j.neuroscience.2018.04.003. 29660443 10.1016/j.neuroscience.2018.04.003

[27] Wijenayake S, Eisha S, Purohit MK, McGowan PO. Milk derived extracellular vesicle uptake in human microglia regulates the DNA methylation machinery : short title: milk-derived extracellular vesicles and the epigenetic machinery. Sci Rep. 2024;14:28630. 10.1038/s41598-024-79724-1. 39562680 10.1038/s41598-024-79724-1 PMC11576889

[28] Zhang H, Shykind B, Sun T. Approaches to manipulating microRNAs in neurogenesis. Front Neurosci. 2013;6:196. 10.3389/fnins.2012.00196. 23335878 10.3389/fnins.2012.00196 PMC3547386

[29] Saliminejad K, Khorram Khorshid HR, Soleymani Fard S, Ghaffari SH. An overview of microRNAs: biology, functions, therapeutics, and analysis methods. J Cell Physiol. 2019;234:5451–65. 10.1002/jcp.27486. 30471116 10.1002/jcp.27486

[30] Liao Y, Du X, Li J, Lönnerdal B Human milk exosomes and their microRNAs survive digestion in vitro and are taken up by human intestinal cells. Mol Nutr Food Res. 2017;61. 10.1002/mnfr.201700082. 10.1002/mnfr.201700082 28688106

[31] Alsaweed M, Lai CT, Hartmann PE, Geddes DT, Kakulas F. Human milk cells contain numerous miRNAs that may change with milk removal and regulate multiple physiological processes. Int J Mol Sci. 2016;17:956. 10.3390/ijms17060956. 27322254 10.3390/ijms17060956 PMC4926489

[32] Zhang Y, Belaid M, Luo X, Daci A, Limani R, Mantaj J, et al. Probing milk extracellular vesicles for intestinal delivery of RNA therapies. J Nanobiotechnology. 2023;21:406. 10.1186/s12951-023-02173-x. 37924132 10.1186/s12951-023-02173-x PMC10623793

[33] Seo M, Anderson G. Gut-amygdala interactions in autism spectrum disorders: developmental roles via regulating mitochondria, exosomes, immunity and microRNAs. Curr Pharm Des. 2019;25:4344–56. 10.2174/1381612825666191105102545. 31692435 10.2174/1381612825666191105102545

[34] Collier SA, Rasmussen SA, Feldkamp ML, Honein MA. National birth defects prevention study. prevalence of self-reported infection during pregnancy among control mothers in the national birth defects prevention study. Birth Defects Res A Clin Mol Teratol. 2009;85:193–201. 10.1002/bdra.20540. 19086018 10.1002/bdra.20540

[35] Brown AS, Meyer U. Maternal immune activation and neuropsychiatric illness: a translational research perspective. Am J Psychiatry. 2018;175:1073–83. 10.1176/appi.ajp.2018.17121311. 30220221 10.1176/appi.ajp.2018.17121311 PMC6408273

[36] Estes ML, McAllister AK. Maternal immune activation: Implications for neuropsychiatric disorders. Science. 2016;353:772–7. 10.1126/science.aag3194. 27540164 10.1126/science.aag3194 PMC5650490

[37] Kentner AC, Bilbo SD, Brown AS, Hsiao EY, McAllister AK, Meyer U, et al. Maternal immune activation: reporting guidelines to improve the rigor, reproducibility, and transparency of the model. Neuropsychopharmacology. 2019;44:245–58. 10.1038/s41386-018-0185-7. 30188509 10.1038/s41386-018-0185-7 PMC6300528

[38] PRGLAC [Task Force on Research Specific to Pregnant Women and Lactating Women], Eunice Kennedy Shriver National Institute of Child Health and Human Development (NICHD) 2018. https://www.nichd.nih.gov/sites/default/files/2018-09/PRGLAC_Report.pdf. Accessed May 28th 2025.

[39] DeRosa H, Caradonna SG, Tran H, Marrocco J, Kentner AC. Got milk? maternal immune activation during the mid-lactational period affects nutritional milk quality and adolescent offspring sensory processing in male and female rats. Mol Psychiatry. 2022;27:4829–42. 10.1038/s41380-022-01744-y. 36056174 10.1038/s41380-022-01744-y PMC9771965

[40] Nascimento AF, Alves GJ, Massoco CO, Teodorov E, Felicio LF, Bernardi MM. Lipopolysaccharide-induced sickness behavior in lactating rats decreases ultrasonic vocalizations and exacerbates immune system activity in male offspring. Neuroimmunomodulation. 2015;22:213–21. 10.1159/000363350. 25139475 10.1159/000363350

[41] Vilela FC, Antunes-Rodrigues J, Elias LL, Giusti-Paiva A. Corticosterone synthesis inhibitor metyrapone preserves changes in maternal behavior and neuroendocrine responses during immunological challenge in lactating rats. Neuroendocrinology. 2013;97:322–30. 10.1159/000346354. 23295343 10.1159/000346354

[42] Aubert A, Goodall G, Dantzer R, Gheusi G. Differential effects of lipopolysaccharide on pup retrieving and nest building in lactating mice. Brain Behav Immun. 1997;11:107–18. 10.1006/brbi.1997.0485. 9299060 10.1006/brbi.1997.0485

[43] Merengueli JA, Kentner AC. Maternal immune activation during the lactational period alters offspring behavior, reproductive development, and immune function in mice. Horm Behav. 2025;173:105776. 10.1016/j.yhbeh.2025.105776. 40543230 10.1016/j.yhbeh.2025.105776 PMC12258413

[44] Edwards PD, Lavergne SG, McCaw LK, Wijenayake S, Boonstra R, McGowan PO, et al. Maternal effects in mammals: broadening our understanding of offspring programming. Front Neuroendocrinol. 2021;62:100924. 10.1016/j.yfrne.2021.100924. 33992652 10.1016/j.yfrne.2021.100924

[45] Golan-Gerstl R, Ben Ya’acov A, Musseri M, Goldenberg R, Chammah Y, Cherki T, et al. Expression profile of MicroRNAs in breast milk of women with inflammatory bowel disease: correlation with disease activity and medical treatments. Inflamm Bowel Dis. 2025;31:912–22. 10.1093/ibd/izae290. 39820274 10.1093/ibd/izae290 PMC11985391

[46] Ren W, La Y, Ma X, Wu X, Guo X, Chu M, et al. Comparative analysis of miRNA expression profiles of yak milk-derived exosomes at different altitudes. Animals. 2025;15:87. 10.3390/ani15010087. 39795030 10.3390/ani15010087 PMC11718820

[47] Wang Y, Fang J, Zeng HF, Zhong JF, Li HX, Chen KL. Identification and bioinformatics analysis of differentially expressed milk exosomal microRNAs in milk exosomes of heat-stressed Holstein cows. Funct Integr Genomics. 2022;22:77–87. 10.1007/s10142-021-00814-8. 34839400 10.1007/s10142-021-00814-8

[48] Connors EJ, Shaik AN, Migliore MM, Kentner AC. Environmental enrichment mitigates the sex-specific effects of gestational inflammation on social engagement and the hypothalamic pituitary adrenal axis-feedback system. Brain Behav Immun. 2014;42:178–90. 10.1016/j.bbi.2014.06.020. 25011058 10.1016/j.bbi.2014.06.020

[49] Núñez Estevez KJ, Rondón-Ortiz AN, Nguyen JQT, Kentner AC. Environmental influences on placental programming and offspring outcomes following maternal immune activation. Brain Behav Immun. 2020;83:44–55. 10.1016/j.bbi.2019.08.192. 31493445 10.1016/j.bbi.2019.08.192 PMC6906258

[50] Zhao X, Mohammed R, Tran H, Erickson M, Kentner AC. Poly (I:C)-induced maternal immune activation modifies ventral hippocampal regulation of stress reactivity: prevention by environmental enrichment. Brain Behav Immun. 2021;95:203–15. 10.1016/j.bbi.2021.03.018. 33766701 10.1016/j.bbi.2021.03.018 PMC8187276

[51] DeRosa H, Caradonna SG, Tran H, Marrocco J, Kentner AC. Milking it for all it’s worth: the effects of environmental enrichment on maternal nurturance, lactation quality, and offspring social behavior. eNeuro. 2022;9:ENEURO.0148-22.2022. 10.1523/ENEURO.0148-22.2022. 35995560 10.1523/ENEURO.0148-22.2022 PMC9417599

[52] Rosenfeld P, Wetmore JB, Levine S. Effects of repeated maternal separations on the adrenocortical response to stress of preweanling rats. Physiol Behav. 1992;52:787–91. 10.1016/0031-9384(92)90415-x. 1409954 10.1016/0031-9384(92)90415-x

[53] Hayley S, Kelly O, Anisman H. Murine tumor necrosis factor-alpha sensitizes plasma corticosterone activity and the manifestation of shock: modulation by histamine. J Neuroimmunol. 2002;131:60–9. 10.1016/s0165-5728(02)00259-x. 12458037 10.1016/s0165-5728(02)00259-x

[54] Kentner AC, James JS, Miguelez M, Bielajew C. Investigating the hedonic effects of interferon-alpha on female rats using brain-stimulation reward. Behav Brain Res. 2007;177:90–9. 10.1016/j.bbr.2006.10.033. 17126922 10.1016/j.bbr.2006.10.033

[55] Drugs and Lactation Database (LactMed). Bethesda (MD): National Library of Medicine (US) 200g; Isoflurane. Bookshelf. https://www.ncbi.nlm.nih.gov/books/NBK501499/. Accessed May 28th 2025.

[56] Lee JJ, Rubin AP. Breast feeding and anaesthesia. Anaesthesia. 1993;48:616–25. 10.1111/j.1365-2044.1993.tb07130.x. 8346780 10.1111/j.1365-2044.1993.tb07130.x

[57] Par Pharmaceutical, Inc, Pitocin [Label]. Par Pharmaceutical, Chestnut Ridge (NY) 2020. https://dailymed.nlm.nih.gov/dailymed/fda/fdaDrugXsl.cfm?setid=6d4b2c25-2e5d-49b5-93bc-2ae8a20916d1&type=display. Accessed May 28th 2025.

[58] Paul HA, Hallam MC, Reimer RA Milk collection in the rat using capillary tubes and estimation of milk fat content by creamatocrit. J Vis Exp. 2015;e53476. 10.3791/53476. 10.3791/53476 PMC4694028 26709708

[59] Wijenayake S, Eisha S, Tawhidi Z, Pitino MA, Steele MA, Fleming AS, et al. Comparison of methods for pre-processing, exosome isolation, and RNA extraction in unpasteurized bovine and human milk. PLoS One. 2021;16:e0257633. 10.1371/journal.pone.0257633. 34591894 10.1371/journal.pone.0257633 PMC8483318

[60] Welsh JA, Goberdhan DCI, O’Driscoll L, Buzas EI, Blenkiron C, Bussolati B, et al. Minimal information for studies of extracellular vesicles (MISEV2023): from basic to advanced approaches. J Extracell Vesicles. 2024;13:e12404. 10.1002/jev2.12404. 38326288 10.1002/jev2.12404 PMC10850029

[61] Morozumi M, Izumi H, Shimizu T, Takeda Y. Comparison of isolation methods using commercially available kits for obtaining extracellular vesicles from cow milk. J Dairy Sci. 2021;104:6463–71. 10.3168/jds.2020-19849. 33714584 10.3168/jds.2020-19849

[62] Naskar S, Narducci R, Balzani E, Cwetsch AW, Tucci V, Cancedda L. The development of synaptic transmission is time-locked to early social behaviors in rats. Nat Commun. 2019;10:1195. 10.1038/s41467-019-09156-3. 30867422 10.1038/s41467-019-09156-3 PMC6416358

[63] Scarborough J, Mueller F, Arban R, Dorner-Ciossek C, Weber-Stadlbauer U, Rosenbrock H, et al. Preclinical validation of the micropipette-guided drug administration (MDA) method in the maternal immune activation model of neurodevelopmental disorders. Brain Behav Immun. 2020;88:461–70. 10.1016/j.bbi.2020.04.015. 32278850 10.1016/j.bbi.2020.04.015

[64] Nguyen HB, Bagot RC, Diorio J, Wong TP, Meaney MJ. Maternal care differentially affects neuronal excitability and synaptic plasticity in the dorsal and ventral hippocampus. Neuropsychopharmacology. 2015;40:1590–9. 10.1038/npp.2015.19. 25598429 10.1038/npp.2015.19 PMC4915255

[65] Bernard PB, Macdonald DS, Gill DA, Ryan CL, Tasker RA. Hippocampal mossy fiber sprouting and elevated trkB receptor expression following systemic administration of low dose domoic acid during neonatal development. Hippocampus. 2007;17:1121–33. 10.1002/hipo.20342. 17636548 10.1002/hipo.20342

[66] Strange BA, Witter MP, Lein ES, Moser EI. Functional organization of the hippocampal longitudinal axis. Nat Rev Neurosci. 2014;15:655–69. 10.1038/nrn3785. 25234264 10.1038/nrn3785

[67] Freeman GH, Halton JH. Note on an exact treatment of contingency, goodness of fit and other problems of significance. Biometrika. 1951;38:141–9. 14848119

[68] Miles J, Shevlin M Applying regression and correlation: a guide for students and researchers. London: Sage; 2001.

[69] Clayton JA. Applying the new SABV (sex as a biological variable) policy to research and clinical care. Physiol Behav. 2018;187:2–5. 10.1016/j.physbeh.2017.08.012. 28823546 10.1016/j.physbeh.2017.08.012

[70] Ordoñes Sanchez E, Bavley CC, Deutschmann AU, Carpenter R, Peterson DR, Karbalaei R, et al. Early life adversity promotes resilience to opioid addiction-related phenotypes in male rats and sex-specific transcriptional changes. Proc Natl Acad Sci USA. 2021;118:e2020173118. 10.1073/pnas.2020173118. 33593913 10.1073/pnas.2020173118 PMC7923376

[71] Hinde K, Skibiel AL, Foster AB, Del Rosso L, Mendoza SP, Capitanio JP. Cortisol in mother’s milk across lactation reflects maternal life history and predicts infant temperament. Behav Ecol. 2015;26:269–81. 10.1093/beheco/aru186. 25713475 10.1093/beheco/aru186 PMC4309982

[72] Sullivan EC, Hinde K, Mendoza SP, Capitanio JP. Cortisol concentrations in the milk of rhesus monkey mothers are associated with confident temperament in sons, but not daughters. Dev Psychobiol. 2011;53:96–104. 10.1002/dev.20483. 20730788 10.1002/dev.20483 PMC3188439

[73] Lu L, Guo H, Peng Y, Xun G, Liu Y, Xiong Z, et al. Common and rare variants of the THBS1 gene associated with the risk for autism. Psychiatr Genet. 2014;24:235–40. 10.1097/YPG.0000000000000054. 25304225 10.1097/YPG.0000000000000054

[74] Paketçi C, Ermiş Ç, Şişman AR, Hız S, Baykara B, Yiş U. Blood neurofilament light chain and thrombospondin-1 levels of patients with autism spectrum disorder. Turk J Med Sci. 2022;52:1041–9. 10.55730/1300-0144.5406. 36326357 10.55730/1300-0144.5406 PMC10388028

[75] Lin, Jun Li F, Ziqi Wang, Zhang T, Lu T, Jiang M, et al. Replication of previous autism-GWAS hits suggests the association between *NAA1, SORCS3*, and *GSDME* and autism in the Han Chinese population. Heliyon. 2023;10:e23677. 10.1016/j.heliyon.2023.e23677. 38234914 10.1016/j.heliyon.2023.e23677 PMC10792458

[76] Dettmer AM, Murphy AM, Guitarra D, Slonecker E, Suomi SJ, Rosenberg KL, et al. Cortisol in neonatal mother’s milk predicts later infant social and cognitive functioning in rhesus monkeys. Child Dev. 2018;89:525–38. 10.1111/cdev.12783. 28369689 10.1111/cdev.12783 PMC6528802

[77] Bagni C, Zukin RS. A synaptic perspective of fragile X syndrome and autism spectrum disorders. Neuron. 2019;101:1070–88. 10.1016/j.neuron.2019.02.041. 30897358 10.1016/j.neuron.2019.02.041 PMC9628679

[78] Joo Y, Benavides DR. Local protein translation and RNA processing of synaptic proteins in autism spectrum disorder. Int J Mol Sci. 2021;22:2811. 10.3390/ijms22062811. 33802132 10.3390/ijms22062811 PMC8001067

[79] Li JY, Daniels G, Wang J, Zhang X. TBL1XR1 in physiological and pathological states. Am J Clin Exp Urol. 2015;3:13–23. 26069883 PMC4446378

[80] Mastrototaro G, Zaghi M, Massimino L, Moneta M, Mohammadi N, Banfi F, et al. TBL1XR1 ensures balanced neural development through NCOR complex-mediated regulation of the MAPK pathway. Front Cell Dev Biol. 2021;9:641410. 10.3389/fcell.2021.641410. 33708771 10.3389/fcell.2021.641410 PMC7940385

[81] Quan Y, Zhang Q, Chen M, Wu H, Ou J, Shen Y, et al. Genotype and phenotype correlations for TBL1XR1 in neurodevelopmental disorders. J Mol Neurosci. 2020;70:2085–92. 10.1007/s12031-020-01615-7. 32524419 10.1007/s12031-020-01615-7

[82] Zhu J, Liu H, Hu Y, Liu J, Dai C, Liang J, et al. Mechanistic insights into retinoic-acid treatment for autism in the improvement of social behavior: Evidence from a multi omics study in rats. Neuropharmacology. 2025;265:110244. 10.1016/j.neuropharm.2024.110244. 39643238 10.1016/j.neuropharm.2024.110244

[83] Kentner AC, Cryan JF, Brummelte S. Resilience priming: translational models for understanding resiliency and adaptation to early life adversity. Dev Psychobiol. 2019;61:350–75. 10.1002/dev.21775. 30311210 10.1002/dev.21775 PMC6447439

[84] Bale TL. Sex differences in prenatal epigenetic programming of stress pathways. Stress. 2011;14:348–56. 10.3109/10253890.2011.586447. 21663536 10.3109/10253890.2011.586447

[85] Lukomska A, Theune WC, Frost MP, Xing J, Kearney A, Trakhtenberg EF. Upregulation of developmentally-downregulated miR-1247-5p promotes neuroprotection and axon regeneration in vivo. Neurosci Lett. 2024;823:137662. 10.1016/j.neulet.2024.137662. 38286398 10.1016/j.neulet.2024.137662 PMC10923146

[86] CDC [Centers for Disease Control]. Breastfeeding special circumstances: illness or conditions and breastfeeding. 2025. https://www.cdc.gov/breastfeeding-special-circumstances/hcp/illnesses-conditions/index.html; influenza: https://www.cdc.gov/breastfeeding-special-circumstances/hcp/illnesses-conditions/flu.html; COVID-19: https://www.cdc.gov/breastfeeding-special-circumstances/hcp/illnesses-conditions/covid-19.html; foodborne and waterborne illness: https://www.cdc.gov/breastfeeding-special-circumstances/hcp/illnesses-conditions/food-water-borne-illness.html. Accessed May 28th 2025.

[87] NICHD Inaugural stakeholder meeting for the prioritization of therapeutic research needs for pregnant, postpartum, and lactating (PPL) persons. 2024. Inaugural Stakeholder Meeting for the Prioritization of Therapeutic Research Needs for Pregnant, Postpartum, and Lactating (PPL) Persons Meeting Summary: https://www.nichd.nih.gov/sites/default/files/inline-files/PPL_Persons_Therapeutic_Needs_Mtng_Summ.pdf. Accessed May 28th 2025.

[88] Tseng PT, Chen YW, Stubbs B, Carvalho AF, Whiteley P, Tang CH, et al. Maternal breastfeeding and autism spectrum disorder in children: A systematic review and meta-analysis. Nutr Neurosci. 2019;22:354–62. 10.1080/1028415X.2017.1388598. 29046132 10.1080/1028415X.2017.1388598

