# Supplementary figures and images for: Investigating milk-derived extracellular vesicles as mediators of maternal stress and environmental intervention
Source: Mol Psychiatry. 2025 Nov 17;31(4):1933–45. doi: 10.1038/s41380-025-03346-w (PMC12674164; doi:10.1038/s41380-025-03346-w)

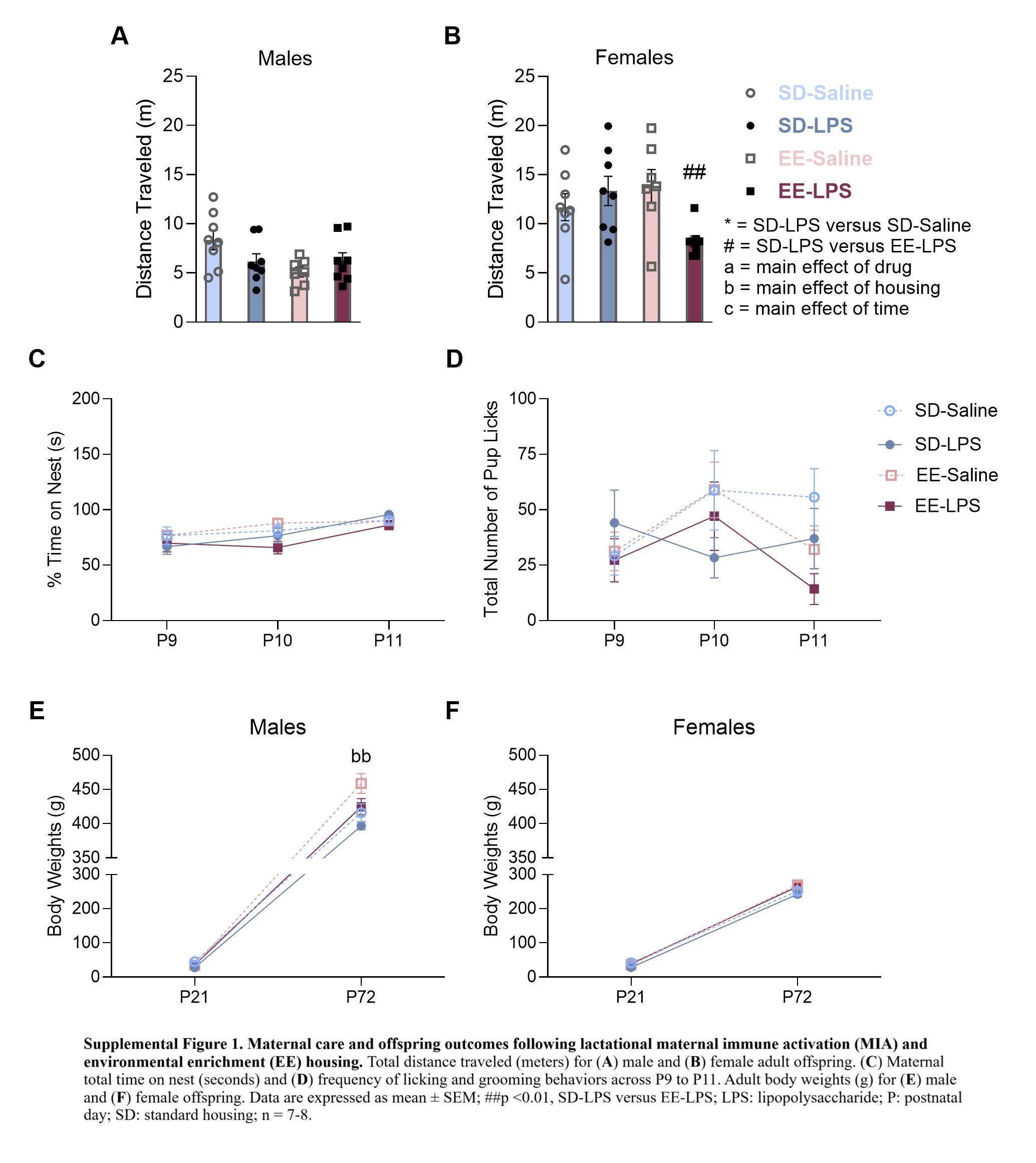

Supplement: Supplementary file 3 — Supplemental Figure 1 [file 41380_2025_3346_MOESM3_ESM.jpg]

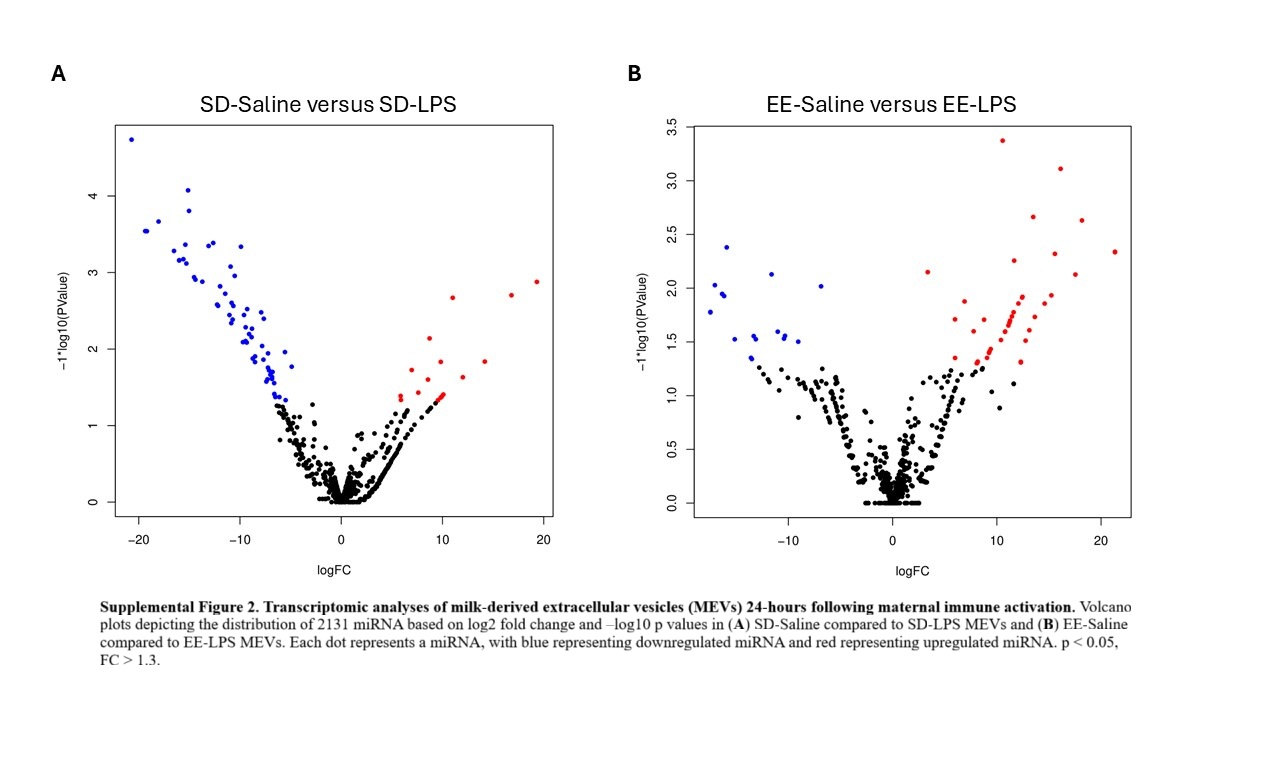

Supplement: Supplementary file 4 — Supplemental Figure 2 [file 41380_2025_3346_MOESM4_ESM.jpg]
