# Supplementary material for: Investigating milk-derived extracellular vesicles as mediators of maternal stress and environmental intervention
Source: Mol Psychiatry. 2025 Nov 17;31(4):1933–45. doi: 10.1038/s41380-025-03346-w (PMC12674164; doi:10.1038/s41380-025-03346-w)
Supplement: Supplementary file 5 — Supplemental Table 2. [file 41380_2025_3346_MOESM5_ESM.docx]

**Supplemental Table 2.** Validation of miRNA-mRNA target predictions from nursing offspring and associated mRNA in the adult hippocampus

| **Group** | **Gene family** |
| --- | --- |
| SD LPS vs. Saline ♂ | *Cd, Clec, Lrrc, Naa, Slc39a, Thbs, Vom2r* |
| SD LPS vs. Saline ♀ | *Sema, Slc, Ube2* |
| EE LPS vs. Saline ♂ | *Cdk/Cdkn* |
| EE LPS vs. Saline ♀ | *Col, Rab, Tmem* |
